# Targeting of phosphatidylserine by monoclonal antibodies augments the activity of paclitaxel and anti-PD1/PD-L1 therapy in the murine breast model E0771

**DOI:** 10.1186/2051-1426-3-S2-P357

**Published:** 2015-11-04

**Authors:** Michael Gray, Jian Gong, Van Nguyen, Takuya Osada, Zachary Hartman, Jeff Hutchins, Bruce Freimark, Kim Lyerly

**Affiliations:** 1 Peregrine Pharmaceuticals Inc., Tustin, CA, USA; 2 Duke University, Durham, NC, USA

## Introduction

The phospholipid lipid phosphatidylserine (PS) normally resides in the inner plasma membrane leaflet in most mammalian cells, including tumor and tumor associated vascular cells. Inducers of cellular stress, such as hypoxia and oxygen radicals encountered in tumors, and treatments by cytotoxic therapies promote PS relocation to the outer leaf of the plasma membrane. In tumors this re-localization allows PS recognition by a number of receptors on myeloid and lymphoid cells in the microenvironment, promoting tumor growth and metastatic disease through the development of an immunosuppressive environment. Currently the PS-targeting antibody bavituximab is being used to treat patients with solid tumors in multiple late-stage clinical trials. Bavituximab's anti-tumor properties are attributed in part through alleviating PS-receptor mediated immunosuppression and assisting in generating an Fc-FcR mediated pro-inflammatory response.

## Methods

Immune competent mice with E0771 induced tumors were administered either paclitaxel, or anti-PD-1/PD-L1 single agent therapy, or in triple combination with PS targeting antibody (ch1N11) to evaluate the efficacy of PS and PD-1/PD-L1 immune checkpoint inhibitors blockade in combination with cytotoxic chemotherapeutics. The levels of PS and PD-L1 expression were also evaluated on E0771 tumor cells following in vitro treatment via FACS analysis.

## Results

Preliminary results in multiple studies demonstrated differential sensitivity to either paclitaxel, or anti-PD-1/PD-L1 single agent therapy while inclusion of ch1N11 triple combination significantly enhanced anti-tumor efficacy over either single agent therapy. Also, FACs analysis demonstrated induction of PS and PD-L1 levels on E0771 cells following *in vitro* treatment with paclitaxel, suggesting that combining paclitaxel with PS and PD-1 targeting antibodies will have greater anti-tumor activity in triple combination treatments. No *in vivo* adverse effects were observed in animals given repeated doses of single or triple combinations.

## Conclusion

These results support combination blockade of PS and PD-1/PD-L1 with paclitaxel to treat breast cancer.

